# Transcriptome and metabolome analysis reveals the effect of flavonoids on flower color variation in *Dendrobium nobile* Lindl.

**DOI:** 10.3389/fpls.2023.1220507

**Published:** 2023-08-23

**Authors:** Yujie Qiu, Chengcheng Cai, Xu Mo, Xinyi Zhao, Lijuan Wu, Fan Liu, Rui Li, Chen Liu, Ji Chen, Mengliang Tian

**Affiliations:** College of Agronomy, Sichuan Agricultural University, Chengdu, China

**Keywords:** *Dendrobium nobile*, flavonoids, anthocyanin, flower variation, transcriptomics

## Abstract

**Introduction:**

*Dendrobium nobile* L. is a rare orchid plant with high medicinal and ornamentalvalue, and extremely few genetic species resources are remaining in nature. In the normal purple flower population, a type of population material with a white flower variation phenotype has been discovered, and through pigment component determination, flavonoids were preliminarily found to be the main reason for the variation.

**Methods:**

This study mainly explored the different genes and metabolites at different flowering stages and analysed the flower color variation mechanism through transcriptome- and flavonoid-targeted metabolomics. The experimental materials consisted of two different flower color phenotypes, purple flower (PF) and white flower (WF), observed during three different periods.

**Results and discussion:**

The results identified 1382, 2421 and 989 differentially expressed genes (DEGs) in the white flower variety compared with the purple flower variety at S1 (bud stage), S2 (chromogenic stage) and S3 (flowering stage), respectively. Among these, 27 genes enriched in the ko00941, ko00942, ko00943 and ko00944 pathways were screened as potential functional genes affecting flavonoid synthesis and flower color. Further analysis revealed that 15 genes are potential functional genes that lead to flavonoid changes and flower color variations. The metabolomics results at S3 found 129 differentially accumulated metabolites (DAMs), which included 8 anthocyanin metabolites, all of which (with the exception of delphinidin-3-o-(2’’’-o-malonyl) sophoroside-5-o-glucoside) were found at lower amounts in the WF variety compared with the PF variety, indicating that a decrease in the anthocyanin content was the main reason for the inability to form purple flowers. Therefore, the changes in 19 flavone and 62 flavonol metabolites were considered the main reasons for the formation of white flowers. In this study, valuable materials responsible for flower color variation in D. nobile were identified and further analyzed the main pathways and potential genes affecting changes in flavonoids and the flower color. This study provides a material basis and theoretical support for the hybridization and molecular-assisted breeding of *D. nobile.*

## 1 Introduction

*Dendrobium nobile* L. is a perennial herb of the genus Dendrobium of the family Orchidaceae. Its medicinal use was first recorded in Shen Nong’s Herbal Classic, an ancient Chinese book written 2,000 years ago. This herb is considered as a top-grade herbal medicine (Mou et al., 2021). Dendrobium contains several bioactive metabolites including: polysaccharides, alkaloids, flavonoids, amongst others (He et al., 2020; Lu et al., 2022). These components offer various health benefits, such as antioxidation, anti-inflammation, and antitumor effects (Lei et al., 2022), improvement of liver damage, and nerve protection (Li D. et al., 2022). Our research group discovered variant strains of *D. nobile* L. that bloom pure white flowers under the same ecological conditions in which the flowers are typically purple and white. Pure white flowers are often associated with qualities such as purity, dignity, holiness, elegance, and cleanliness. Consumers are particularly attracted to varietal varieties with white flowers due to their ornamental qualities. The color of flowers is considered to be one of the key characteristics of ornamental plants, and it plays a crucial role in breeding programs. The formation of flower color in most plants is primarily influenced by flavonoid compounds, particularly anthocyanins (Sun et al., 2021; Zhang et al., 2020). Upon analyzing the dendrobine content of the two strains of Dendrobium nobile, it was observed that the stems of the white-flowered strains exhibited a significantly higher dendrobine content compared to the purple-flowered strains. This finding not only highlights the enhanced ornamental value of the white-flowered strains, but also signifies their greater medicinal worth. Consequently, the white-flowered strains possess a higher economic value due to their increased medicinal potential.

Flavonoids are a group of natural compounds with a C6-C3-C6 carbon skeleton. These compounds can be divided into subclasses based on the position of the connection of the phenyl ring and the oxygen-containing γ-pyrone ring and the oxidation status and degree of saturation of the heterocyclic ring. These subclasses include flavones, flavonols, flavanones, flavanonols, anthocyanidins, flavanols, and isoflavones (Shamsudin et al., 2022). More than 9000 types of plant flavonoids have been identified and isolated to date. The flavonoids commonly found in foods include apigenin, luteolin (Taheri et al., 2021), quercetin (Yi et al., 2021), myricetin (Wang et al., 2020), hesperetin (Yeh et al., 2022), taxifolin, catechin (Munteanu and Apetrei, 2022), and daidzein. Modern pharmacological studies have shown that flavonoids exert antitumor, antibacterial, antifungal and antiviral effects and have been widely used in the research and development of new drugs and in the food and cosmetic industries. Flavonoids not only provide plants with attractive color attributes to attract insects, birds and other animals for pollination and seed dispersal (Trunschke et al., 2021) but also participate in plant homeostasis as osmotic regulators, providing plants with the ability to resist biotic and abiotic stresses (Wang et al., 2022; Zhuang et al., 2023).

More than 700 types of anthocyanins have been identified in nature, and these include cyanidin (Cy), delphinidin (Dp), pelargonidin (Pg), peonidin (Pn), petunidin (Pt), and malvidin (Mv), which are six common anthocyanin pigments (Peng et al., 2021; Lin et al., 2023). The biosynthesis of anthocyanins is actually part of the late stage of flavonoid synthesis. The entire biosynthetic pathway is catalyzed by the starting amino acid phenylalanine (Phe) to form 4-coumarin CoA through phenyl amine ammonia-lyase (PAL), cinnamate-4-hydroxylase (C4H) and 4-coumaroil-CoA ligase (4CL). Under the catalysis of chalcone synthase (CHS), yellow chalcone is produced, and chalcone isomerase (CHI) and flavanone 3-hydroxylase (F3H) then catalyze the formation of dihydroflavonols. Under the catalysis of flavonoid 3’-hydroxylase (F3’H) and flavonoid 3’,5’-hydroxylase (F3’ 5’H), a dihydroflavonol forms the precursors of synthetic anthocyanins, namely, dihydroquercetin and dihydromyricetin. Colorless anthocyanidin is then formed by the action of dihydroflavonol reductase (DFR), and the colored anthocyanin is formed by the catalysis of leucoanthocyanidin oxygenase/anthocyanidin synthase (LDOX/ANS) followed by UDP-glucosyl-flavonoid-3-O-glycosyltransferase. Under the action of UFGT, anthocyanin forms glycosidic bonds with one or more units of galactose, rhamnose, or glucose, among others, and is ultimately converted into stable anthocyanins (Li et al., 2020; Samkumar et al., 2021; Mohammed and Khan, 2022). Research has shown that the overexpression or silencing of enzyme-encoded genes can affect the accumulation and changes in flavonoids, including anthocyanins, in plants and may affect color changes (Sánchez-Cabrera et al., 2021; Ruan et al., 2022).

The study of plant flavonoids and anthocyanin synthesis pathways is one of the most popular research topics at present, but the flower color variation mechanism of *D. nobile* and the biosynthesis and regulation processes of flavonoids have not been reported. With the development of science and technology, scientific research has utilized sequencing technology for the comprehensive analysis of differentially expressed genes (DEGs) and differentially accumulated metabolites (DAMs) in plant samples through the use of transcriptomics and metabolomics (Li et al., 2020; Rao et al., 2021; Li H. et al., 2022). This study constitutes the first comprehensive analysis of the transcription and metabolism of two species of *D. nobile* flowers, constructed a flavonoid biosynthesis pathway of *D. nobile*, and identified the regulatory genes that may be involved in the flavonoid synthesis and flower coloration of *D. nobile* flowers. This study is helpful for revealing the mechanism underlying the effect on *D. nobile* and Orchidaceae from the perspective of molecular biology and to improve the active ingredients of Dendrobium flavonoids from the perspective of genetics.

## 2 Materials and methods

### 2.1 Source and identification of plant materials

Purple flower (PF) and white flower (WF) varieties of *D. nobile* were discovered and collected from Luzhou, Sichuan (longitude: 105°53′5″, latitude: 28°40′42″) in April 2017. These varieties were propagated and grown for 3 years in the greenhouse at the Teaching Experimental Base of Sichuan Agricultural University, Chengdu (longitude: 103°51′38″, latitude: 30°42′19″). The WF variety was proven to be a variant strain of *D. nobile* L. based on morphology, main medicinal components and ITS2 molecular marker identification.

The components and contents of dendrobine in the PF and WF varieties were identified by gas chromatography. The CTAB method was used for the extraction of DNA from flower tissue of the WF and PF varieties, and the primer pair *ITS2-F* and *ITS2-R* was used to clone ITS2 sequences by PCR. *DNAMAN* software was used for sequence alignment analysis.

### 2.2 Determination of flavonoids and anthocyanin content

During flower development, the flowers of the PF strain undergo a color change from green to white with purple pigmentation, while the flowers of the WF strain only become white. In the early stage of floral bud development, when the green bud reaches a length of approximately 1 cm, there are slight but not obvious differences between the two buds. This period is referred to as S1. As the bud continues to develop and grows to a length of approximately 2 cm, noticeable color differences begin to appear on the surface of the flowers. This period is referred to as S2. These differences continue to increase, leading to more significant color variations. When the flowers first open, the phenotypic color difference between the two flowers has reached its maximum, and this period is referred to as S3.

S3 samples from PF and WF varieties, 200 mg of each sample were triturated with 5 ml of PBS solution in a centrifuge tube. The flavonoid and anthocyanin contents were measured using the Plant Anthocyanin Test Kit and Fla ELISA Test Kit, respectively. The multiple comparison of contents between different strains were determined with Duncan method using SPSS software version 22.0 (SPSS Inc., Chicago, IL, USA). Data were analyzed using a t-test and P < 0.05 was considered significant.

### 2.3 Transcriptome sequencing

For transcriptome studies, S1, S2, and S3 samples of both PF and WF varieties were utilized. Each sample consisted of 3 biological replicates, with each replicate comprising 5 independent individual sources of samples. The cDNA libraries were sequenced on the Illumina sequencing platform by Metware Biotechnology Co., Ltd. (Wuhan, China). RNA degradation and contamination were monitored on 1% agarose gels. The RNA purity was checked using a NanoPhotometer^®^ spectrophotometer (IMPLEN, CA, USA). The RNA concentration was measured using a Qubit^®^ RNA Assay Kit and a Qubit^®^2.0 Fluorometer (Life Technologies, CA, USA). The RNA integrity was assessed using the RNA Nano 6000 Assay Kit and the Bioanalyzer 2100 system (Agilent Technologies, CA, USA). A total amount of 1 μg of RNA per sample was used as the input material for the RNA sample preparations. Sequencing libraries were generated using the NEBNext^®^ Ultra™ RNA Library Prep Kit for Illumina^®^ (NEB, USA) following the manufacturer’s recommendations, and index codes were added to classify the sequences. The clustering of the index-coded samples was performed on a cBot Cluster Generation System using TruSeq PE Cluster Kit v3-cBot-HS (Illumina) according to the manufacturer’s instructions. After cluster generation, the library preparations were sequenced on an Illumina platform, and 150-bp paired-end reads were generated. Fastp v 0.19.3 was used to filter the original data, mainly to remove reads with adapters. All subsequent analyses were based on clean reads.

### 2.4 Differential expression analysis

The genome of *Dendrobium officinale* was regarded as a reference for alignment and annotation analysis. HISAT v2.1.0 was used to construct the index and compare clean reads to the reference genome. FeatureCounts v1.6.2 and StringTie v1.3.4d were used for new gene prediction and to calculate the gene alignment and FPKM values. DESeq2 v1.22.1 and edgeR v3.24.3 were used to analyse the differential expression between the PF and WF groups, and the P value was corrected using the Benjamini and Hochberg method. The corrected P value and absolute log_2_FC (fold change) values were used as thresholds to indicate significant differences in expression. The enrichment analysis was performed based on the hypergeometric test. For the KEGG analysis, the hypergeometric distribution test was performed with the unit of pathway. GO analysis was performed based on the GO terms. The protein interaction analysis of DEGs was based on the STRING database of known and predicted protein-protein interactions. For the species existing in the database, a network was constructed by extracting the target gene list from the database. Otherwise, Diamond v0.9.24.125 was used to compare the target gene sequence with the selected reference protein sequence, and the network was then established based on the known interactions of the selected reference species. Gsea-3.0.jar and WGCNA v1.69 were used for gene set enrichment analysis and weighted gene coexpression network analysis, respectively.

### 2.5 Flavonoid-targeted metabolomics analysis

For flavonoid targeted metabolomics studies, S3 samples of both PF and WF varieties were utilized. Each sample consisted of 3 biological replicates, with each replicate comprising 5 independent individual sources of samples. The biological samples were freeze-dried in a vacuum freeze-dryer (Scientz-100F) and crushed using a mixer mill (MM 400, Retsch) with a zirconia bead for 1.5 min at 30 Hz. One hundred milligrams of lyophilized powder was dissolved in 1.2 ml of 70% methanol solution, and the sample was placed in a refrigerator at 4°C overnight. Following centrifugation at 12000 rpm for 10 min, the extracts were filtered (SCAA-104, pore size of 0.22 μm, ANPEL, Shanghai, China). UPLC-MS/MS analysis was conducted by Metware Biotechnology Co., Ltd. (Wuhan, China). Based on the company’s database, the qualitative analysis of substances was conducted according to the secondary spectrum information. During the analysis, the isotopic signals, the repeated signals containing K^+^, Na^+^, and NH4^+^ ions, and the repeated signals of fragment ions corresponding to other substances with higher molecular weights were removed. Metabolite quantification was completed by triple quadrupole mass spectrometry in the multiple reaction monitoring mode. The identified metabolites were annotated using the KEGG Compound database, and the annotated metabolites were then mapped to the KEGG Pathway database. Pathways with significantly regulated metabolites mapped to this database were then used for metabolite set enrichment analysis (MSEA), and their significance was determined based on hypergeometric test p values. The univariate analysis and orthogonal partial least squares-discriminant analysis (OPLS-DA) results were used to screen the DAMs between the PF and WF varieties. The difference was considered significant only if the variable importance in projection (VIP) value was ≥1 or the absolute log_2_FC was ≥1.

### 2.6 Integration analysis of DEGs and DAMs

The enriched KEGG pathways identified by transcriptomics and metabolomics were used to draw a bubble diagram. The quantitative values of the DEGs and DAMs in all the samples were used for correlation analysis, and correlation coefficients greater than 0.80 and p values less than 0.05 were selected for nine-quadrant analysis. All correlation results obtained for the DEGs and DAMs were selected to draw a correlation cluster heatmap. The O2PLS model was used for integration analysis of two datasets.

### 2.7 qRT-PCR analysis

Total RNA in floral tissues of the PF and WF varieties was extracted using the TRIzol reagent (Invitrogen, Carlsbad, CA, USA), and reverse transcription and cDNA synthesis were performed with a Vazyme kit. cDNA was used as the template to measure gene expression. Three independent biological replicates were used for each quantitative experiment, and the expression level of each gene was calculated based on the 2^-ΔΔCt^ algorithm. The specific primers used in this study are listed in **Supplementary Table 1**.

## 3 Results

### 3.1 Identification of the WF variety

**Figure 1:**
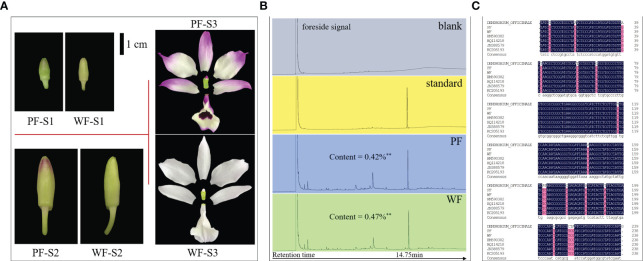
Floral morphology, dendrobine content and genomic identification of the PF and WF varieties. **(A)** Color differences at different flower development stages. **(B)** Determination of the dendrobine content by gas chromatography. **(C)** ITS2 sequence alignment. HM590382, HQ114218, JN388579 and KC205193 were the local IDs of *D. nobile* collected in the NCBI database. Three biological replicates were used for dendrobine determination. ^**^ *p*<0.01.

No visual phenotypic differences were found between the PF and WF varieties with the exception of the color of floral tissues (**Supplementary Figure 1**). In the flowers of the WF variety, all tissues that should have exhibited a purple appearance were white. With extension of the flower development period, the difference in flower color became more obvious (**Figure 1A**). Therefore, the content of dendrobine, the main medicinal component of *D. nobile*, was identified and determined in the PF and WF varieties by HPLC. The results showed that the dendrobine content in the WF variety was extremely significantly higher than that in the PF variety, with values of 0.47% and 0.42%, respectively. The retention time of the main peak of the PF and WF solutions was consistent with that of the standard solution, indicating that the WF variety could be a variation population of the PF variety, which are classified as *D. nobile* (**Figure 1B**).

To further confirm this conclusion, an analysis was also performed at the genomic level. By amplifying and sequencing the ITS2 sequence, a specific DNA fragment of *D. nobile*, the ITS2 sequence in the WF variety was found to be completely consistent with that in the PF variety and other published *D. nobile* sequences. Genomic DNA was extracted from *D. officinale*, conducted a ITS2 sequence alignment analysis and found that it was not consistent with the sequence of *D. nobile* but exhibited high sequence coverage and consistency (**Figure 1C**). All the results indicated that the WF variety is a new species subtype of *D. nobile*.

### 3.2 Measurement of color indices and flavonoid contents

**Figure 2:**
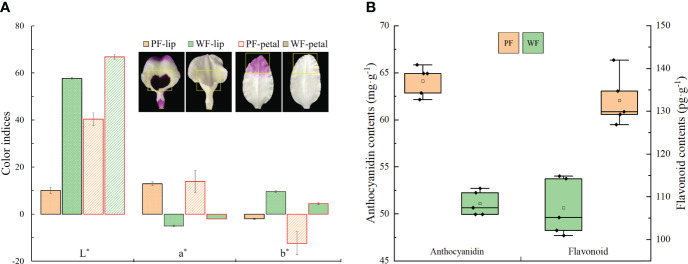
Color indices and contents of anthocyanins and flavonoids in the PF and WF varieties. **(A)** Color indices of different floral tissues. **(B)** Determination of the anthocyanin and flavonoid contents. The error bars represent the SEs of three biological replicates.

To identify the flower characteristics of the PF and WF varieties, the color indices of the petals and lip at S3 were measured with a colorimeter. The analysis of the color indices of lips or petals revealed that the L* value (brightness) and b* value (yellowness) of the WF variety were always higher than those of the PF variety, and the opposite result was obtained for the a* value (redness), which proves that the flowers of the WF variety were markedly brighter and that the flowers of the PF variety were markedly darker (**Figure 2A**). Furthermore, the accumulation of flavonoids and anthocyanins in the PF and WF varieties at S3 was analyzed (**Figure 2B**). The results show that the contents of flavonoids or anthocyanins in the PF variety were higher than those of the WF variety. The contents of flavonoids and anthocyanins in the WF variety were decreased by 18.9% and 20.3%, respectively, compared with those in the PF variety. These results suggest that a low content of flavonoids and anthocyanins may be the main factor that causes the variation in flower color from purple to white.

### 3.3 Analysis of DAMs

To clarify the specific metabolic mechanism of flower color variation, flowers at the S3 stage were used to detect flavonoid metabolites, and 286 metabolites were detected based on the UPLC-MS/MS detection platform and the company’s database (**Supplementary Table 2**). The quality control samples were evaluated by coefficient of variation (CV) analysis, overlap analysis of the total ion current (TIC) and principal component analysis (PCA), which showed that the data exhibited good stability, repeatability and reliability (**Supplementary Figures 2–4**). The clustering heatmap of all metabolites showed that the metabolite levels showed significant differences between the PF and WF varieties. The number of metabolite that were decreased in the WF variety compared with the PF variety was greater the number that were increased in the WF variety compared with the PF variety (**Supplementary Figure 5**).

**Figure 3:**
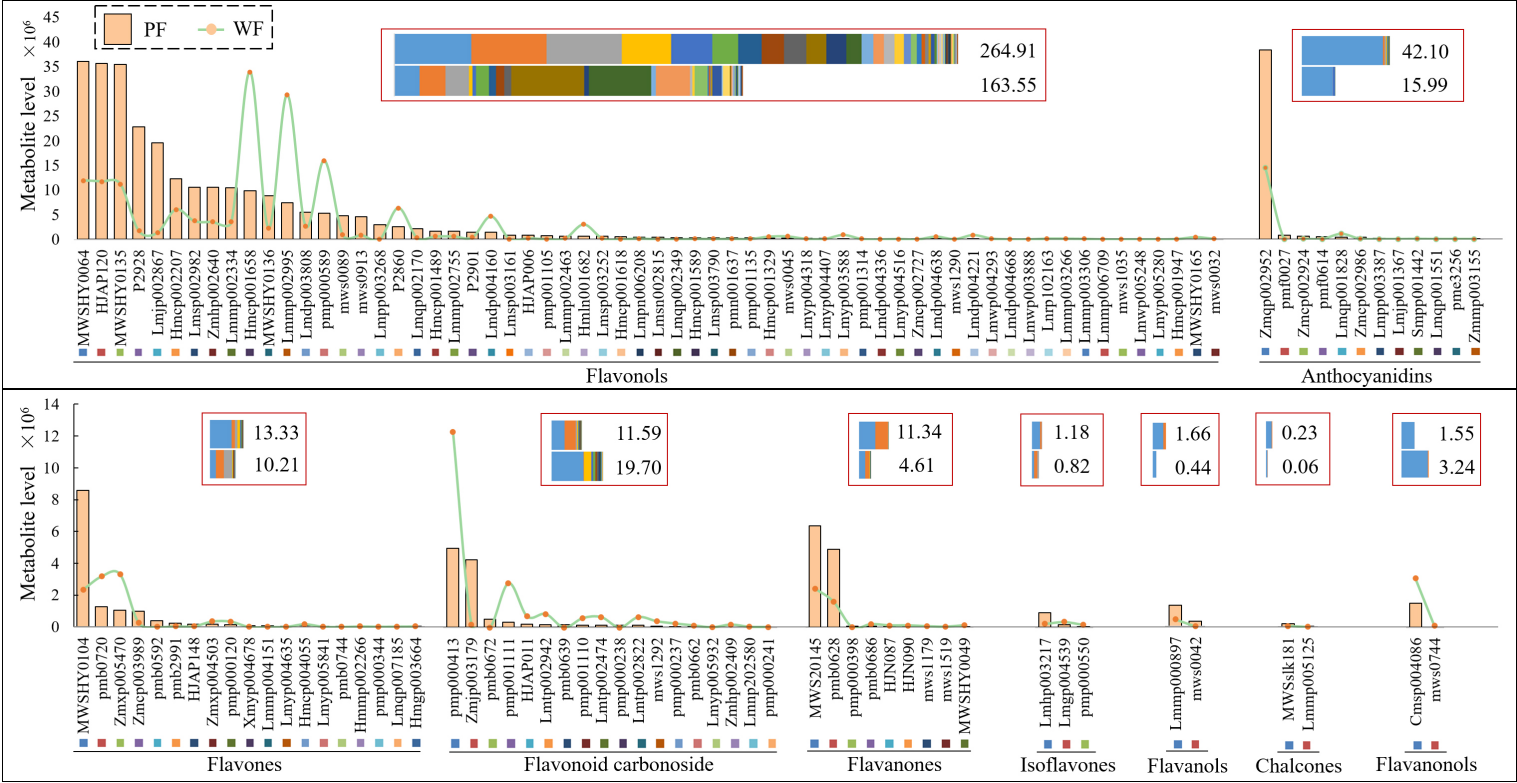
Heatmaps of flavonoid metabolites in two different colored flowers of *D. nobile*. PF, purple flower; WF, white flower. Each individual graph represents different subclasses of flavonoids.

Based on the fold change obtained from the univariate analysis, 129 DAMs (78 downregulated and 51 upregulated DAMs) were screened and analyzed. Admittedly, only a few metabolites were annotated in the CPD and KEGG databases (**Supplementary Figure 5** and **Supplementary Table 3**). Nine types of DAMs were obtained from the secondary classification of flavonoid-targeted substances, and among these, flavonols accounted for the largest number of DAMs (62 metabolites). Delphinidin-3-O-rutinoside-7-O-glucoside (Zmqp002952), an anthocyanin, was found at the highest level among all metabolites and exhibited the largest proportion among the total level of anthocyanin metabolites (greater than 92%). In addition, the total level of all metabolite types, with the exception of flavonoid carbonoside and flavanonols, in the PF variety was always higher than that in the WF variety. Notably, some DAMs showed extremely large differences, and these DAMs are likely to be the main reason for the variation in flower color. The levels of DAMs, including mws1035, Lmpp003268, Lmyp005280, Lmdp004336, Lmwp003888, Lmdp004668, Lmqp002349, Lmwp005248, Zmcp002727, Xmyp004678, pmb0592, Lmmp004151, pmf0027, Lmjp001367, pme3256, pmf0614, Lmpp003387, Lmqp001551, pmb0639, pmb0672 and mws0042, were markedly higher in the PF variety than in the WF variety, and Hmgp003664, mws0744 and MWSHY0049 showed high levels in the WF variety (**Figure 3**).

### 3.4s Overview of the transcriptomic analysis

**Figure 4:**
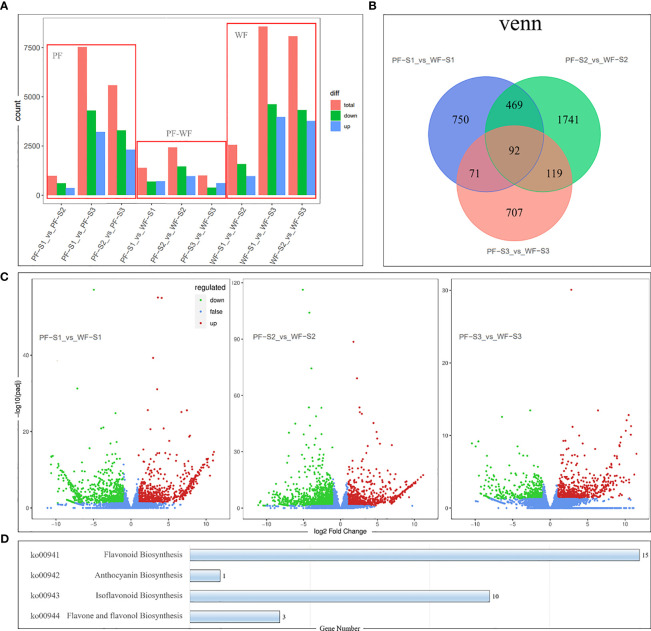
Analysis of differentially expressed genes (DEGs) derived from comparative transcriptomics among the different colors and periods of experimental *D. nobile* materials. **(A)** Numbers of total, downregulated, and upregulated DEG. **(B)** Venn diagram displaying the up/downregulated DEGs. Three comparison groups were established to reveal the flavonoid accumulation differences: PF-S1 vs. WF-S1, PF-S2 vs. WF-S2, and PF-S3 vs. WF-S3. **(C)** Volcano map diagram of DEGs in the different colored flowers of *D. nobile.* Red dots indicate upregulated DEGs, green dots indicate downregulated DEGs, and blue dots indicate genes that did not show differential expression. **(D)** Number of DEGs involved in the flavonoid biosynthesis (ko00941), anthocyanin biosynthesis (ko00942), isoflavonoid biosynthesis (ko00943) and flavone and flavonol biosynthesis (ko00944) pathways.

The gene expression profiles of the PF and WF varieties at three flower development stages were studied by transcriptome sequencing. A total of 34747 genes, including 12856 new genes, were detected, and the gene expression and annotation information of these genes are listed in **Supplementary Table 4**. Differential expression analysis between the sample groups was conducted by DESeq2 to obtain a set of DEGs between two biological conditions. This study identified 9 sets of DEGs: the PF and WF groups contained the S1-S2, S2-S3 and S1-S3 sets, respectively, and the PF-WF group contained the PFS1-WFS1, PFS2-WFS2 and PFS3-WFS3 sets (**Figure 4A**). The PF and WF groups represent the differential gene expression changes in the two varieties during flower development. The results showed that the number of DEGs increased during the developmental process in both the PF and WF varieties, and the gene expression patterns of both groups were similar. However, the number of DEGs in the WF group was higher than that in the PF group, indicating that the formation of white flowers is more complex.

In addition, the three sets of DEGs of the PF-WF group can directly reflect the difference in gene expression between the two varieties. The PFS1-WFS1, PFS2-WFS2 and PFS3-WFS3 sets consisted of 1382, 2421 and 989 DEGs, respectively (**Figure 4B**). Among these DEGs, the total number of downregulated genes was greater than that of upregulated genes, and the greatest difference in gene expression was found in the PFS2-WFS2 set (**Figure 4C**). Pathway annotation analysis of the DEGs was conducted based on the KEGG database. The main enrichment pathways of the PF-WF group showed that the DEGs were mainly concentrated in various metabolic pathways (**Supplementary Figure 7**). Specifically, 15, 1, 10 and 3 DEGs were found to be involved in the flavonoid biosynthesis (ko00941), anthocyanin biosynthesis (ko00942), isoflavonoid biosynthesis (ko00943) and flavone and flavonol biosynthesis (ko00944) pathways, respectively (**Figure 4D**), and the specific gene information is listed in **Supplementary Table 5**.

### 3.5 Integrated analysis of the metabolome and transcriptome

In this study, three sets of DEGs in the PF-WF group and DAMs at S3 were used to perform the integration analysis. The KEGG enrichment analysis results are shown in **Supplementary Figure 8**, and the gene and metabolite information of the three groups used in the integration analysis is provided in **Supplementary Table 6**. The quantitative values of genes and metabolites in all the samples were used as the basic data for the correlation analysis. In general, consistent or opposite patterns of DEGs and DAMs indicate their correlation (**Supplementary Figure 9**), which also shows that the changes in metabolites may be under positive or negative regulation of genes, and the information on DEGs and DAMs with their correlations is listed in **Supplementary Table 7**. To further screen the important variables in the two omics, all DEGs and DAMs were selected to establish the O2PLS model, and the variables with high correlation and weight in different data groups were preliminarily judged through the loading map (**Supplementary Figure 10**). The DEGs and DAMs involved in the ko00941, ko00942, ko00943 and ko00944 pathways in the O2PLS model were also screened simultaneously (**Supplementary Table 8**). However, because the metabolites detected in this study are rarely annotated in the current KEGG or GO database, DAMs screened in the O2PLS model results are not necessarily the most important variable. It is necessary to make further inferences based on the different levels of DAMs and the potential functions reported in the current literature.

**Figure 5:**
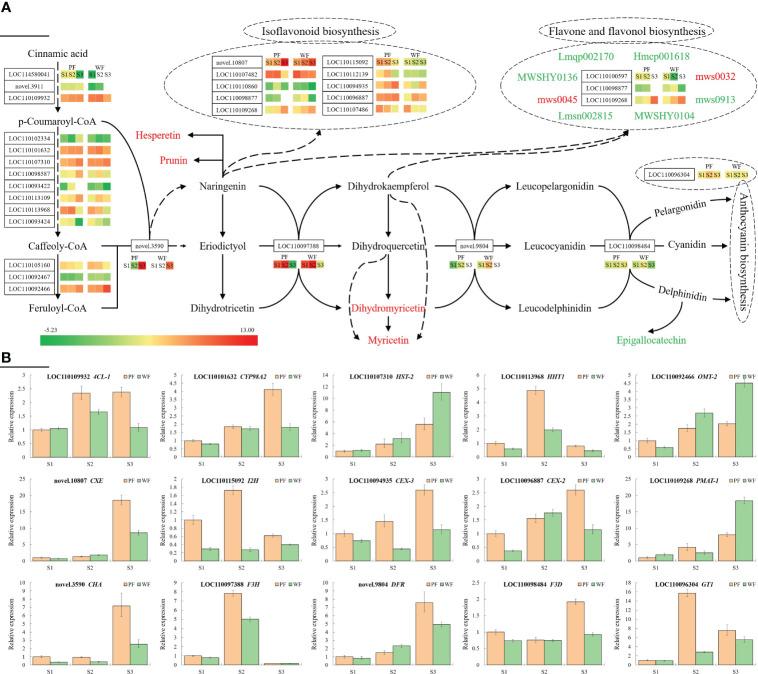
Metabolic regulatory network and gene expression analysis of the flavonoid synthesis pathway in *D. nobile*. **(A)** Central metabolic network dominating the gene expression and metabolite flux differences in the overall flavonoid biosynthesis pathway in the two *D. nobile* materials. Key regulatory genes catalyzing each enzymatic step are marked in solid font, and their expression differences in the three pairwise comparisons are reflected by the comparison of the calibrated FPKM values of each unigene. For a unigene, a positive value indicates upregulated expression, whereas a negative value indicates downregulated expression. The color change in the gene expression heatmap from green to red indicates low to high expression. The changes in crucial metabolic nodes are represented by different colors: red indicates upregulation, and green indicates downregulation. Important derivatives of the isoflavonoid biosynthesis, flavone and flavonol biosynthesis, and anthocyanin biosynthetic pathways are collectively integrated into the three dashed boxes marked. **(B)** Relative expression levels of 15 genes involved in the flavonoid biosynthesis pathway at three stages analyzed by qRT-PCR.

To more intuitively analyse the cause of the difference, the DEGs and DAMs in the flavonoid synthesis-related pathway are summarized in **Figure 5A**. It was hypothesized that these highly expressed genes have a higher possibility of causing color differences. To investigate and verify the expression pattern of these highly expressed genes at three flowering stages, 15 genes, as shown in **Figure 5B**, were used for qRT-PCR assay. The results indicated that, with the exception of *DFR* (novel.9804), the expression patterns of the majority of genes were in line with the transcriptome. In the transcriptomic analysis, the expression of *DFR* was not detected in the two materials at the S3 stage, whereas the qRT-PCR results showed that the expression of this gene was highest at the S3 stage. In addition, no significant difference was found in the expression of most genes at S1 between the PF and WF varieties, which may also explain why the color difference was not obvious at S1. Consistent with the flower development process, the differences in these genes at S2 and S3 became more obvious. Among these genes, the expression of *4CL-1* (LOC110109932), *CYP98A2* (LOC110101632), *HHT1* (LOC110113968), *CXE* (novel.10807), *I2H* (LOC110115092), *CEX-3* (LOC110094935), *CEX-2* (LOC110096887), *CHA* (novel.3590), *CYP98A2* (LOC110097388), *DFR* (novel.9804), *F3D* (LOC110098484) and *GT1* (LOC110096304) was higher in the PF variety, whereas the other three genes, *HST-2* (LOC110107310), *OMT-2* (LOC110092466) and *PMAT-1* (LOC110109268), were more highly expressed in the WF variety. These DEGs are believed to be the main reason for the difference in metabolites and flower color between the PF and WF varieties.

## 4 Discussion

This study was based on white-flowered mutant strains of *D. nobile* found during the resource investigation process. Cultivation-based comparison found that the white-flowered strains were completely consistent with normal purple-flowered strains and only identified differences in the flower color during the flowering period. Therefore, based on the differences in flower color, this study constructed the biosynthetic pathway of flavonoids in *D. nobile* flowers through a multiomics analysis of the transcriptome and metabolome and provided a large number of candidate genes and related data. In this study, a triple quadrupole-linear ion trap mass spectrometer (Q TRAP), AB4500 Q TRAP UPLC/MS/MS system equipped with an ESI Turbo ion-spray interface, was used to detect and annotate 286 flavonoid metabolites and provides the first targeted detection of flavonoids in the flowers of *D. nobile*. The study found that differences in flavonoid compounds, particularly anthocyanins such as cyanidin and paeoniflorin, are the primary cause of the variation in flower color among white-flowered strains of *D. nobile*. The results from similar studies on color regulation in different plants, including *Dendrobium officinale* (Zhan et al., 2020), *Saccharum officinarum* (Rao et al., 2021), *Camellia japonica* (Fu et al., 2021), *Paeonia × suffruticosa* (Luo et al., 2021), and *Brassica napus* (Jia et al., 2021), support these findings. In order to maintain stability, anthocyanins often exist in the form of monoglycosides. Several monoglycoside derivatives of cyanidins and peonidins were identified in the purple petals of PF. Notably, cyanidin-3-O-galactoside, peonidin-3,5-O-diglucoside, peonidin-3-O-(6’’-O-p-coumaroylglucoside)-5-O-glucoside, cyanidin-3-O-(2’’-O-xylosyl)galactoside, and cyanidin-3-O-sophorotrioside were either found or detected in significantly higher concentrations compared to the white petals. These findings suggest that these specific anthocyanins may play crucial roles in enhancing the deep purple color of PF petals (Fu et al., 2021; Shao et al., 2022).

*D. nobile*, a member of the Orchidaceae family, is a stunning ornamental plant with bright and colorful flowers. The peaceful and elegant feeling that white flowers of *D. nobile* evoke makes it a favorite among consumers. The discovery of white-flowered *D. nobile* has enriched the germplasm resources of the plant, opening up the possibility for the creation of Dendrobium and Orchidaceae plants with more flower colors. *D. nobile* is a valuable medicinal plant, and its stems and flowers are being used for various purposes. Purple flowers contain rich anthocyanins, which are natural pigments that can be used as food additives to enhance the color of food. Additionally, these pigments have potential health benefits (Arruda et al., 2021; Sun et al., 2021; Bocker and Silva, 2022). However, there are differences in the accumulation of compounds in different colored petals. (Yu et al., 2022);. White flowers have been found to reduce the synthesis of anthocyanins while increasing the accumulation of substances such as hesperidin, myricetin, apigenin, and kaempferol, which presents an opportunity for the extraction of these natural substances from *D. nobile*. This may play the same role or provide new guidance in obtaining more Gardenia yellow pigment from *G. jasminoides* var. *radicans* Makikno (Wu et al., 2020), extracting flavonoids from flowers (Mlcek et al., 2021), and using myricetin and kaempferol as anticancer substances (Rahmani et al., 2023).

This study aimed to analyse the differences in flower development and color accumulation between purple- and white-flowered lines of *D. nobile*. Transcriptome sequencing was conducted during three key periods of flower color development, and a differential gene set was obtained through differential expression analysis between the sample groups. The study found that the number of downregulated genes among the DEGs was higher than the number of upregulated genes in the PF variety. This study focused on key enzyme genes involved in flavonoid synthesis pathways, including *DnCHS*, *DnF3’*H, *DnDFR*, and *DnGT1*. The study also used qRT-PCR to verify the expression levels of these genes during flower development and coloration. The expression levels of key enzyme genes increased during the gradual coloration process of purple flowers, and the gene expression levels were much higher in purple flowers than in white flowers at the same developmental period. The main reason for the difference in color is the differential expression of key enzyme genes, which is consistent with previous studies on different plant species (Cai et al., 2022; Lee et al., 2023);. Synthase functional genes play a crucial catalytic role in the biosynthesis of flavonoids and anthocyanins (Wang et al., 2021; Kou et al., 2023; Ma et al., 2023). Mutations resulting from loss of synthase function can affect the biosynthesis of flavonoids and anthocyanins, and thus, plants carrying these mutations produce light or colorless tissues (Sakuta et al., 2021; Gonda et al., 2023).

Flower color polymorphism within a population is valuable for comprehending the fundamental evolutionary processes that create and sustain trait variation. It can serve as a model for studying plant microevolution by examining the variation in flower color within a population (Sapir et al., 2021). Most flowering plants depend on animals for pollination, and their color, scent and shape play a crucial role in attracting pollinators (Baguette et al., 2020; van der Kooi et al., 2021b), and there is evidence that flower color may be regulated and evolve in response to pollinator visual systems (van der Kooi et al., 2021a). Pollinators can actually perceive not only pigments in the visible range but also pigments that absorb ultraviolet radiation (Stoddard et al., 2020). The visible pigments in flowers that are mainly responsible for flower color are anthocyanins that are visible to the naked eye, whereas ultraviolet pigments are usually flavonols that also originate from the flavonoid synthesis pathway (Stavenga et al., 2021; Todesco et al., 2022). Although the content of anthocyanins in white flowers is minimal, the content of flavonols, the precursors of anthocyanins, is notably higher in white flowers than in purple flowers. This difference in the flavonol content can have an impact on the communication signals between plants and pollinators, which can affect the attraction of pollinators to flowers (Lüthi et al., 2022; Binaghi et al., 2023).

## 5 Conclusion

In this study, metabolome and transcriptome analyses were used to identify key flavonoids and candidate genes responsible for the formation of purple flowers in *D. nobile*. A total of 129 flavonoids were detected among the DAMs: 62 flavonols, 12 anthocyanidins, 19 flavones, 18 flavonoid carbonoside, 9 flavanones, 3 isoflavones, 2 flavonols, 2 chalcones, and 2 flavanonols. Anthocyanins, especially delphinidin-3-O-rutoside-7-O-glucoside, may be the main components that affect the different colors of flowers. Moreover, several synthase genes (e.g., *DnF3’H*, *DnDFR*, and *DnGT1*) in the flavonoid biosynthesis pathway were identified as candidate regulators contributing to anthocyanin biosynthesis or transport in *D. nobile* flowers. Flavonoids with antioxidant and free radical scavenging properties derived from medicinal plants will be more favored by consumers than synthetic chemicals. The findings of this study will provide valuable information and new insights for further investigations of the regulatory network underlying the accumulation of flavonoids in *D. nobile*.

## Data availability statement

The datasets presented in this study can be found in online repositories. The names of the repository/repositories and accession number(s) can be found below: NCBI Bioproject accession number: PRJNA975329.

## Author contributions

FL, JC, and MT conceived and designed the experiments. YQ and XZ collected the plant samples. YQ, XM and CC performed the experiments. LW, RL and CL analyzed the data. YQ and CC wrote the paper. All authors contributed to the article and approved the submitted version.
