# Supplementary figures and images for: Transcriptome and metabolome analysis reveals the effect of flavonoids on flower color variation in Dendrobium nobile Lindl
Source: Front Plant Sci. 2023 Aug 23;14:1220507. doi: 10.3389/fpls.2023.1220507 (PMC10481954; doi:10.3389/fpls.2023.1220507)

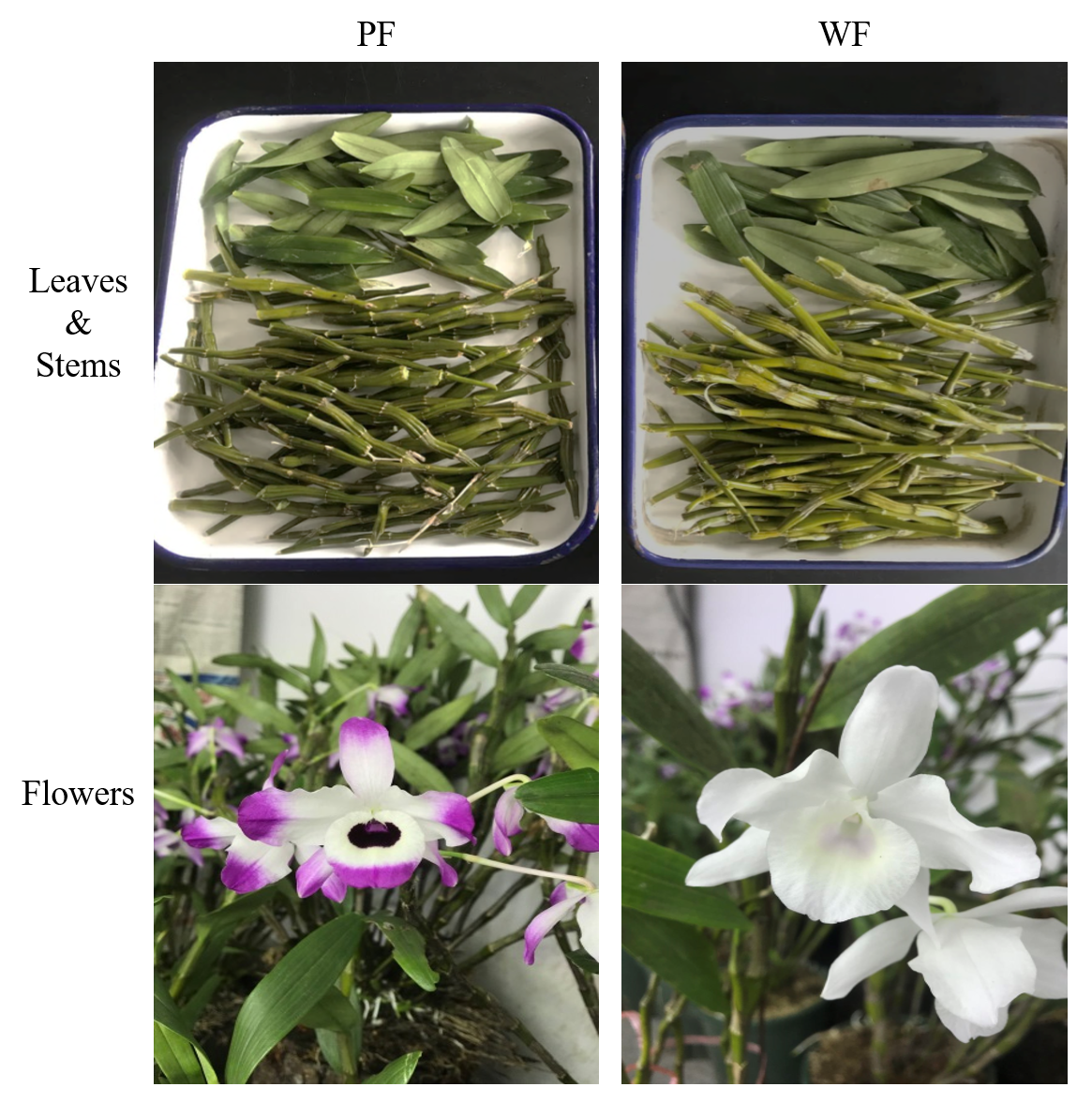

Supplement: Supplementary file 2 [file DataSheet_2.zip › Additional files- figures/SF 1.png]

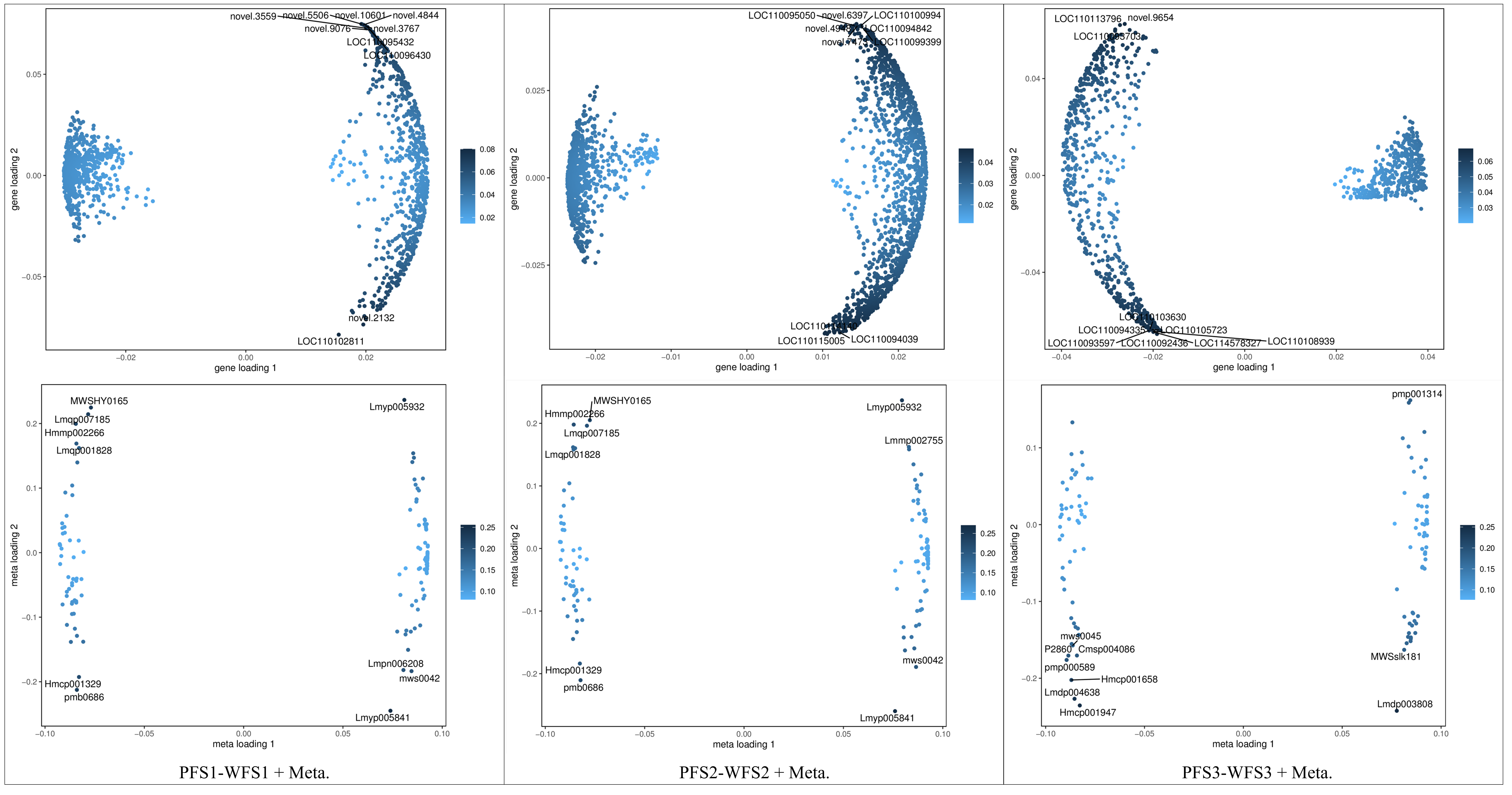

Supplement: Supplementary file 2 [file DataSheet_2.zip › Additional files- figures/SF 10.png]

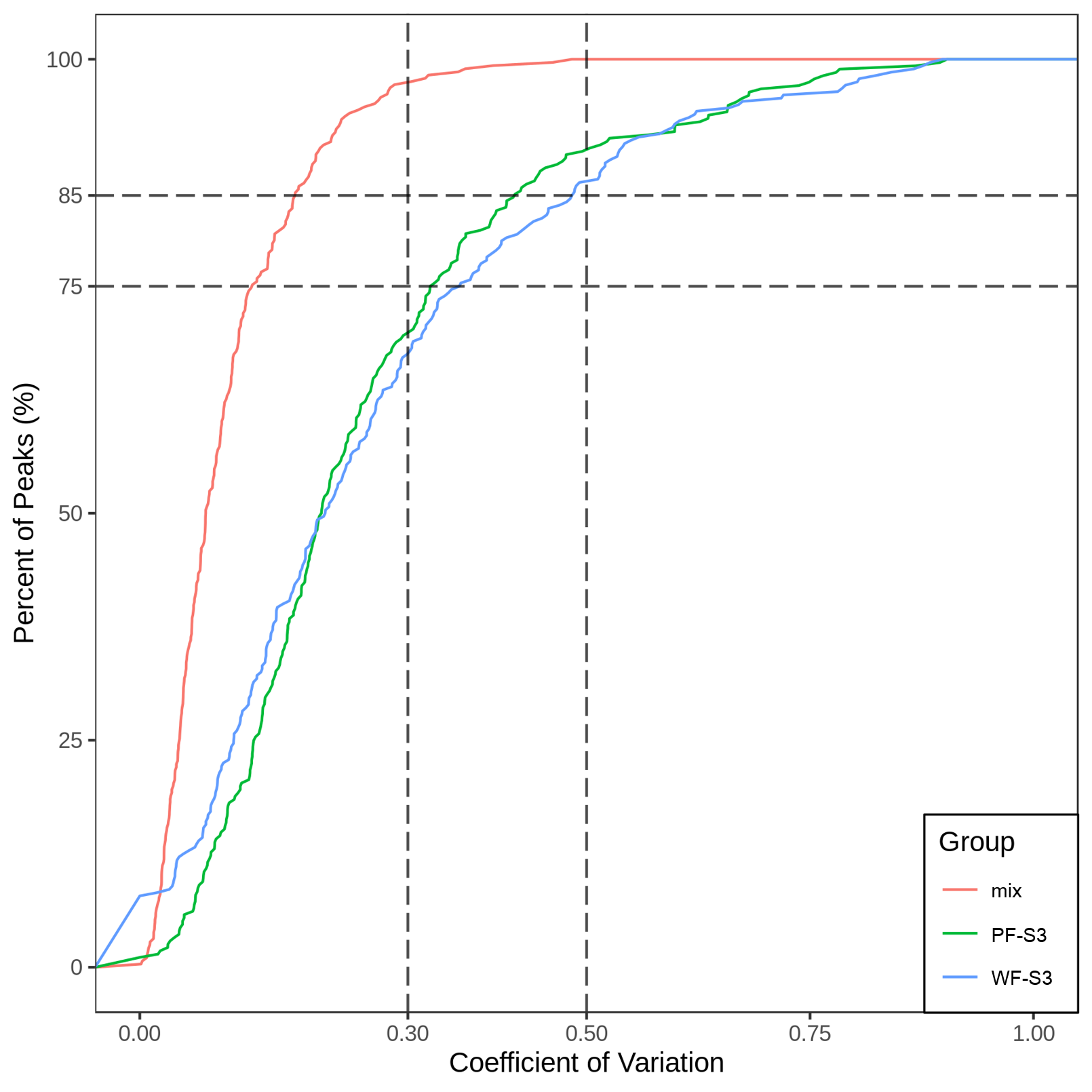

Supplement: Supplementary file 2 [file DataSheet_2.zip › Additional files- figures/SF 2.png]

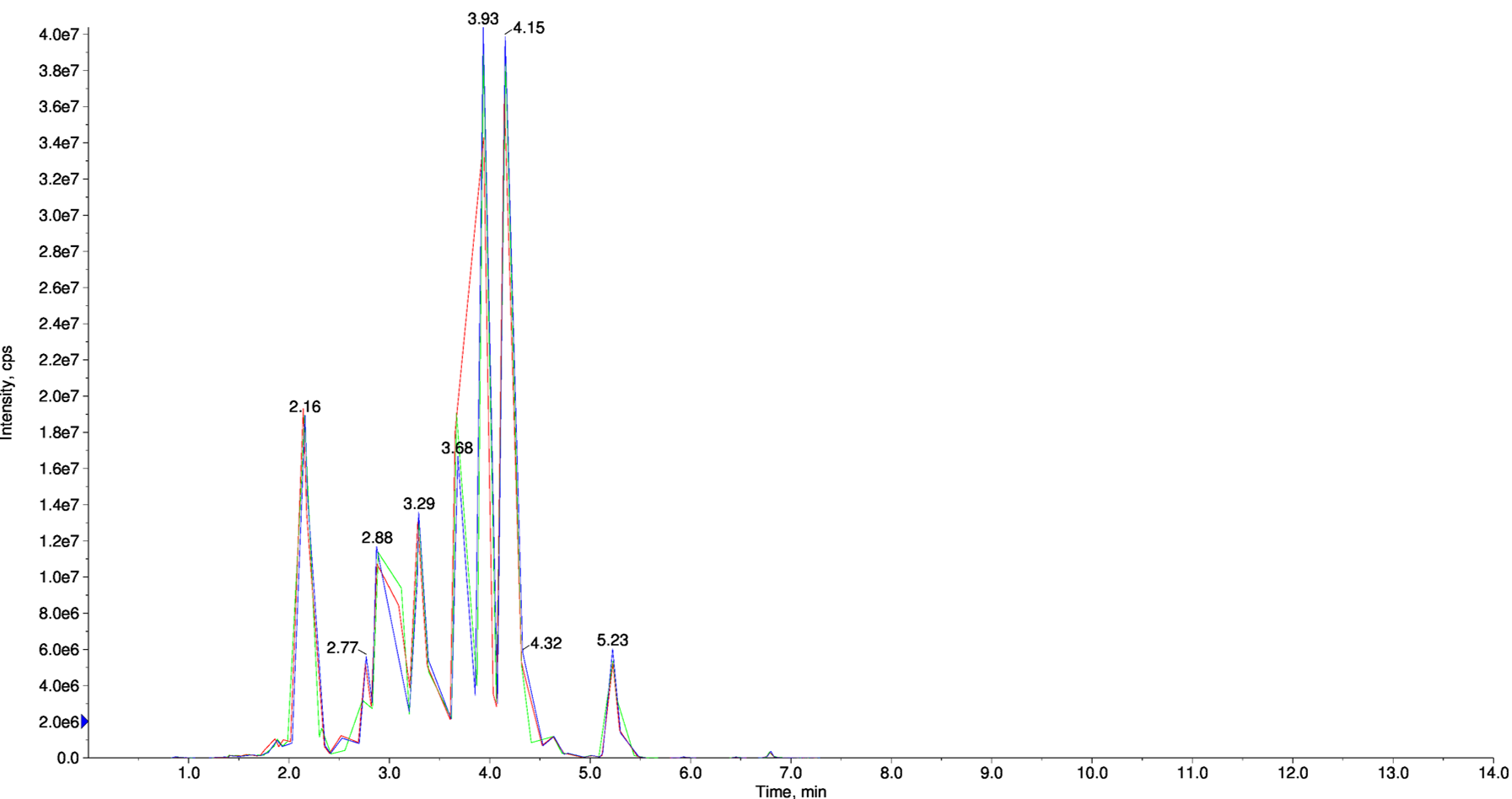

Supplement: Supplementary file 2 [file DataSheet_2.zip › Additional files- figures/SF 3.png]

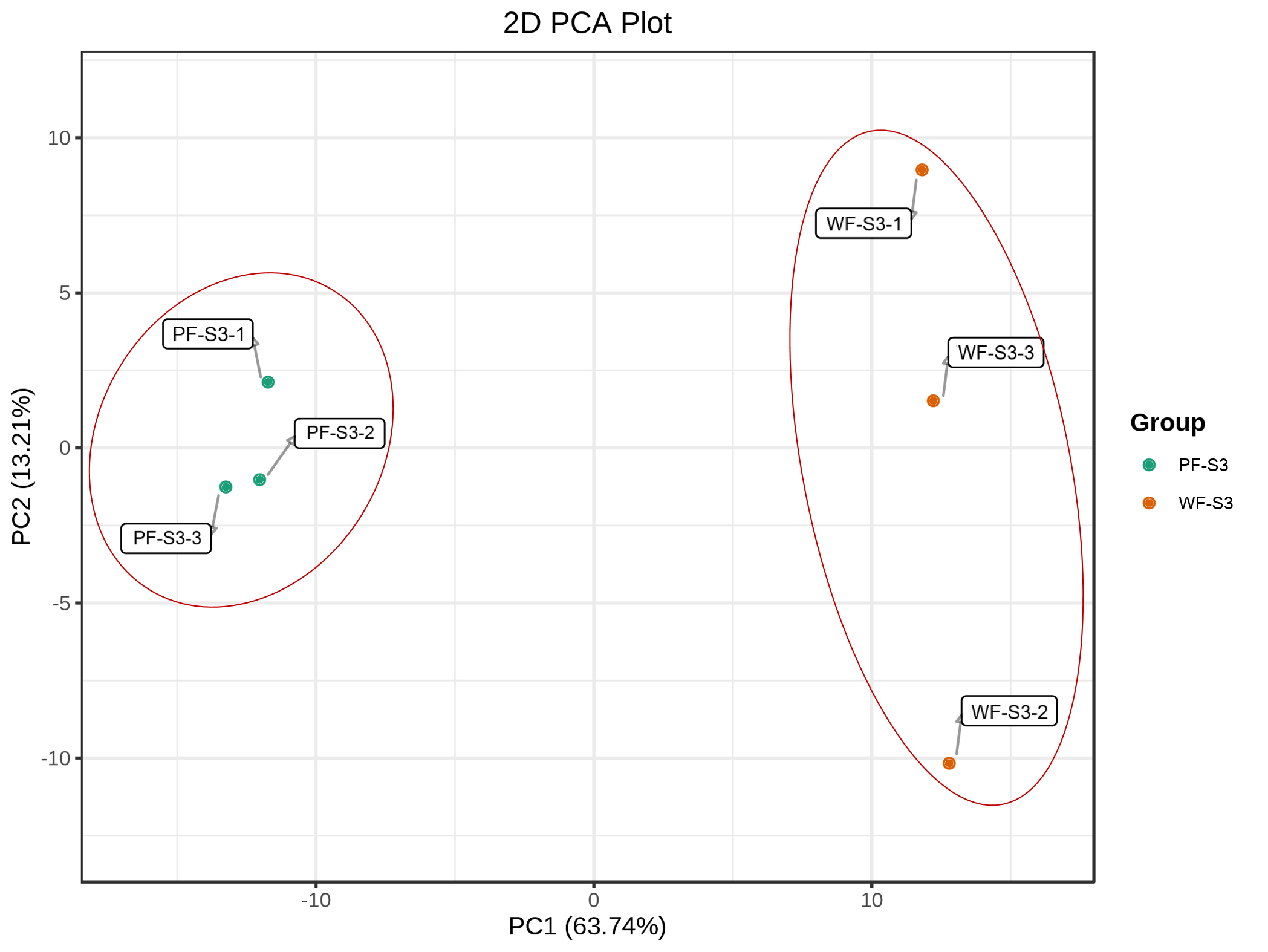

Supplement: Supplementary file 2 [file DataSheet_2.zip › Additional files- figures/SF 4.png]

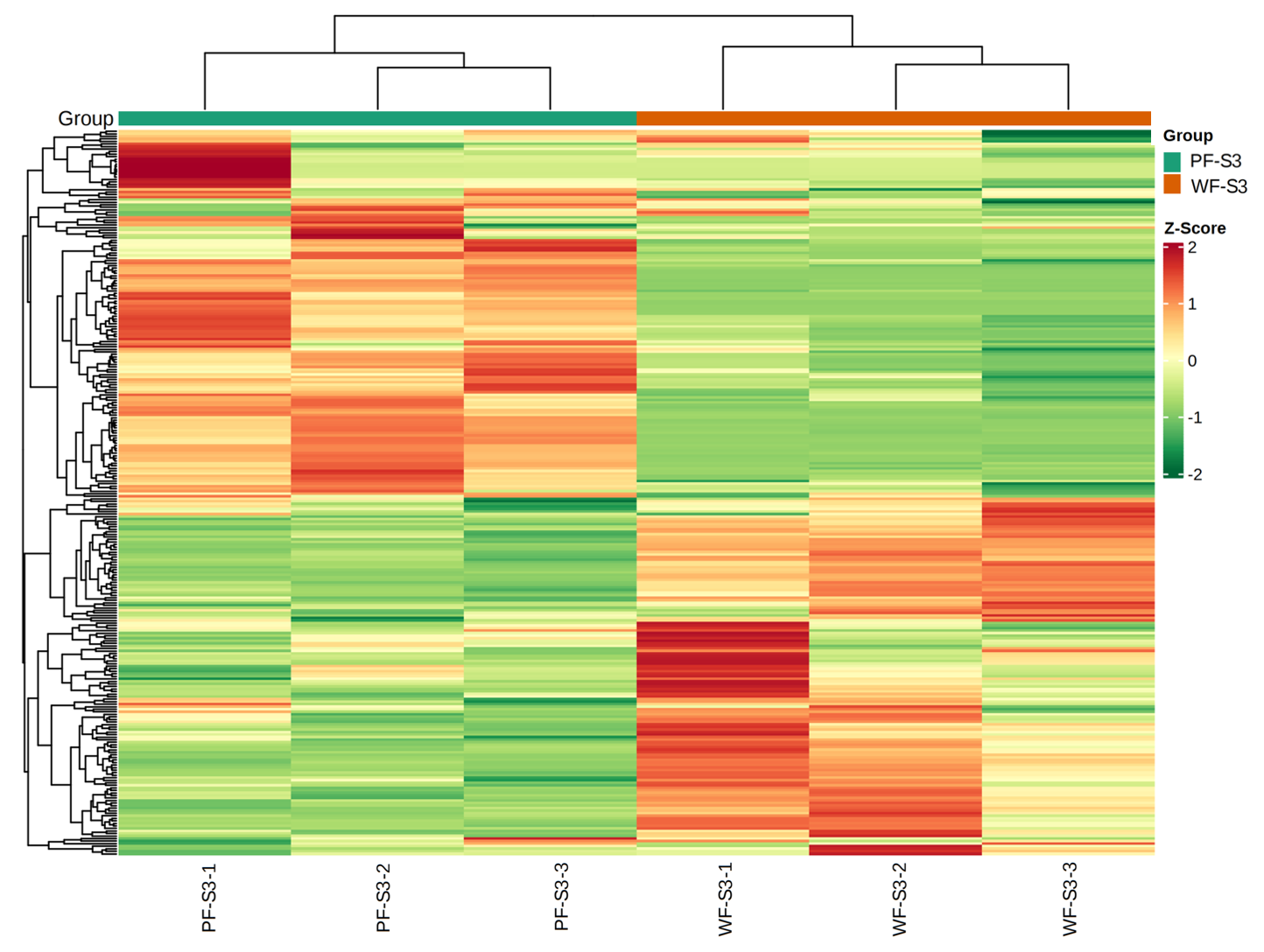

Supplement: Supplementary file 2 [file DataSheet_2.zip › Additional files- figures/SF 5.png]

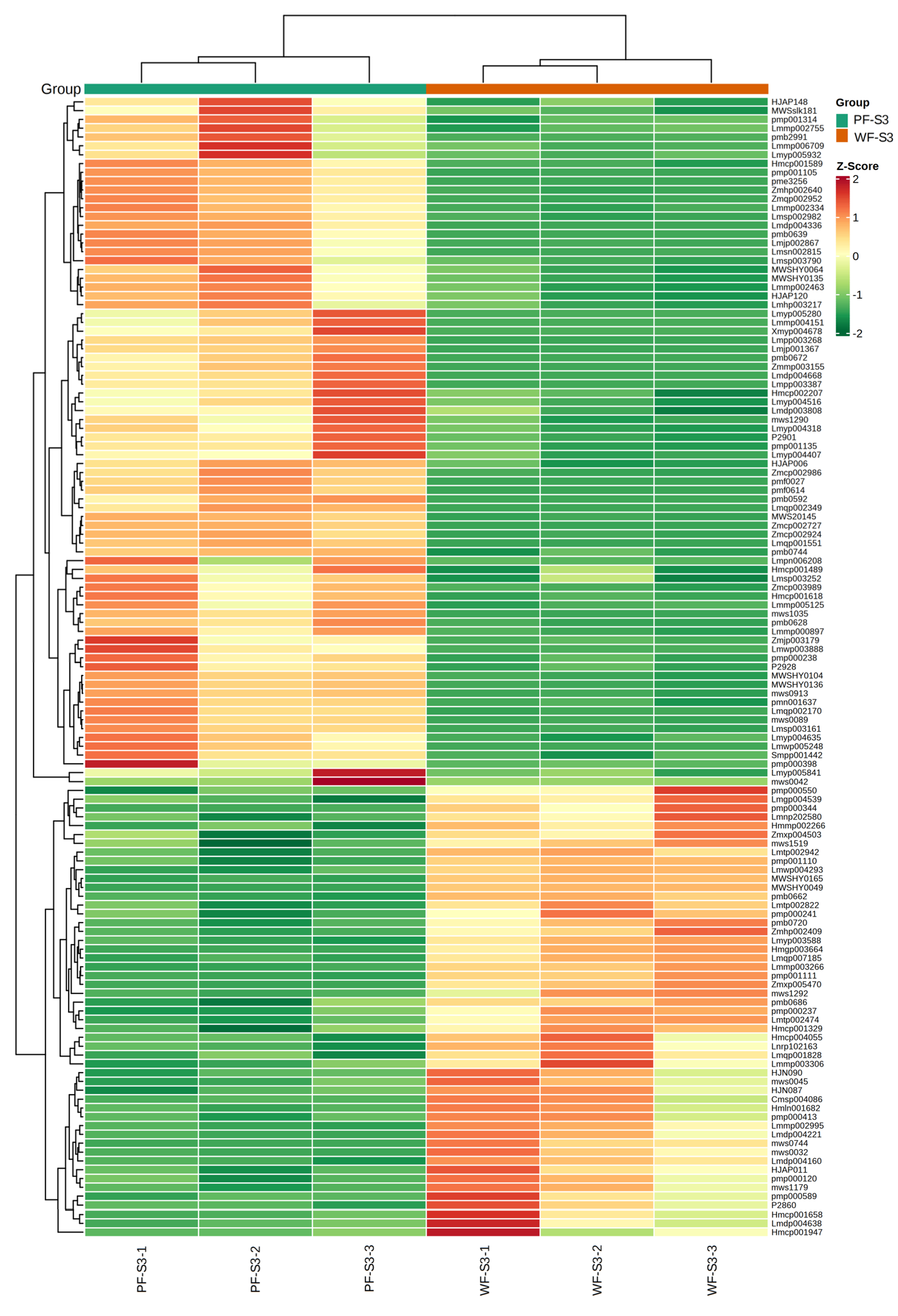

Supplement: Supplementary file 2 [file DataSheet_2.zip › Additional files- figures/SF 6.png]

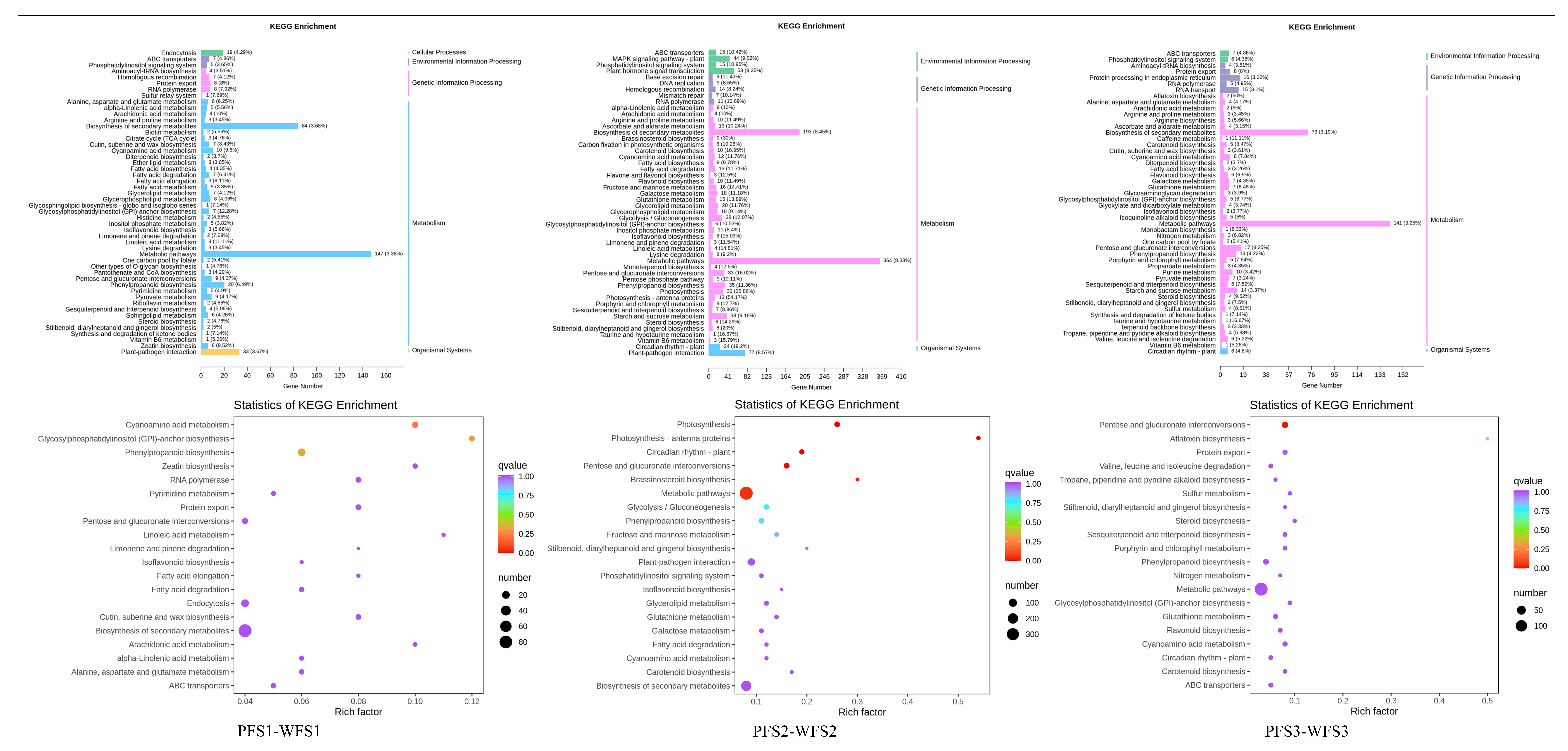

Supplement: Supplementary file 2 [file DataSheet_2.zip › Additional files- figures/SF 7.png]

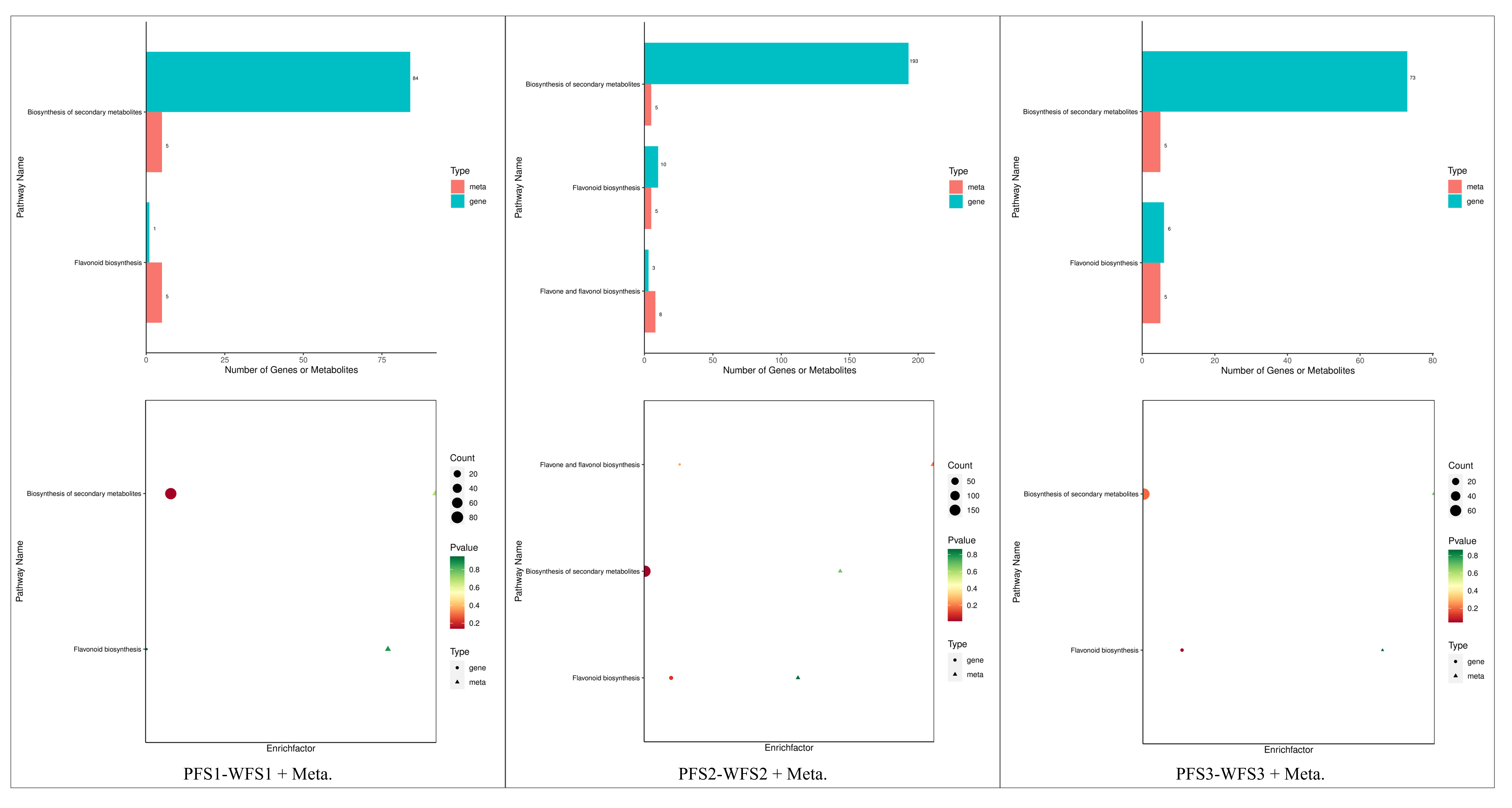

Supplement: Supplementary file 2 [file DataSheet_2.zip › Additional files- figures/SF 8.png]

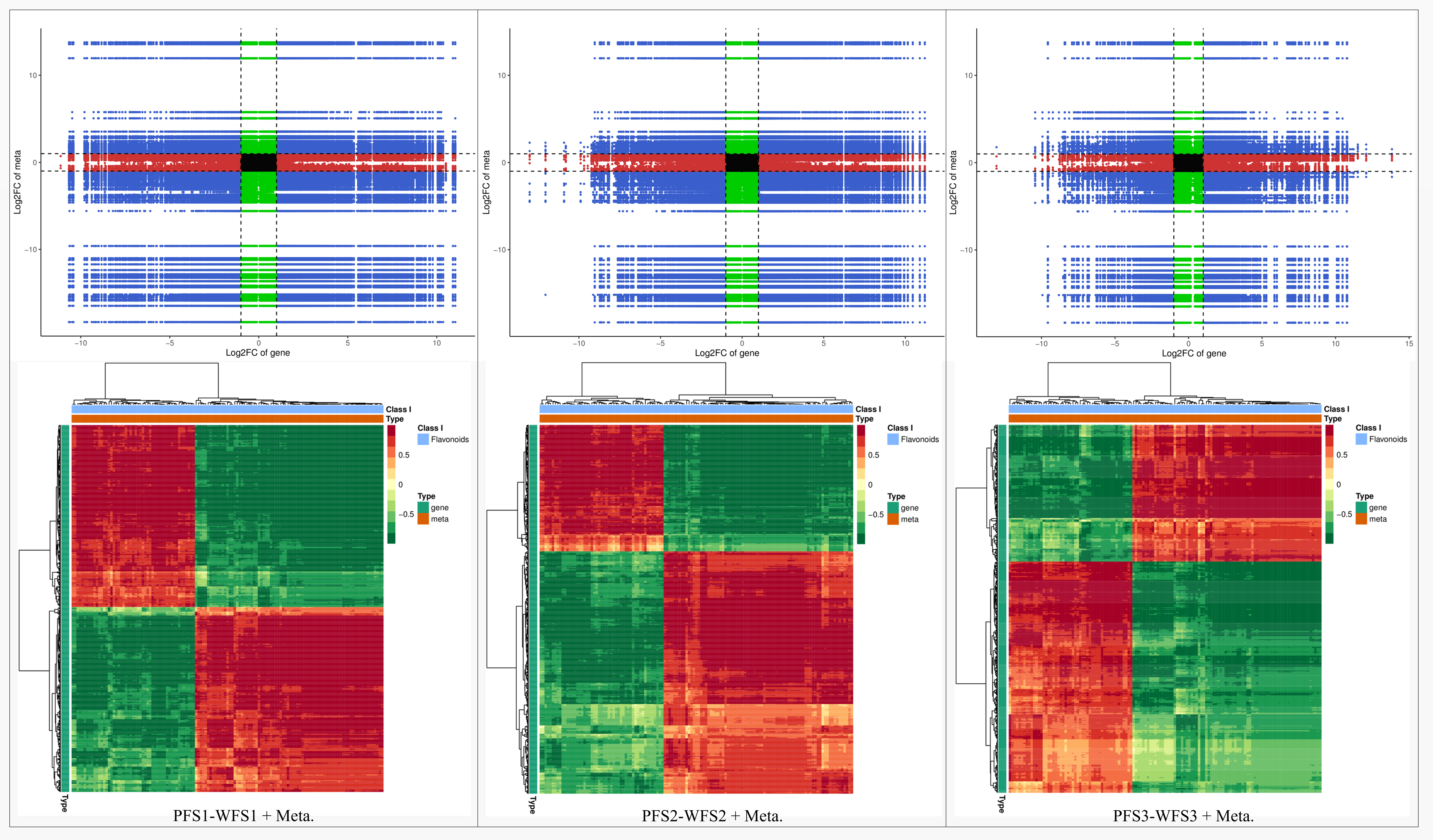

Supplement: Supplementary file 2 [file DataSheet_2.zip › Additional files- figures/SF 9.png]
